# Serum albumin and white matter hyperintensities

**DOI:** 10.21203/rs.3.rs-3822513/v1

**Published:** 2024-01-11

**Authors:** Natalie Zahr, Adolf Pfefferbaum

## Abstract

Urine albumin, high in kidney disease, predicts cardiovascular incidents and CNS white matter hyperintensity (WMH) burdens. Serum albumin – a more general biomarker which can be low in several disorders – including kidney and liver disease, malnutrition, and inflammation – also predicts cardiovascular events and is associated with cognitive impairment in several clinical populations; relations between serum albumin and WMH prevalence, however, have rarely been evaluated. In a sample of 160 individuals with alcohol use disorder (AUD), 142 infected with HIV, and 102 healthy controls, the hypothesis was tested that lower serum albumin levels would predict larger WMH volumes and worse cognitive performance irrespective of diagnosis. After considering traditional cardiovascular risk factors (e.g., age, sex, body mass index (BMI), nicotine use, hypertension, diabetes) and study-relevant variables (i.e., primary diagnoses, race, socioeconomic status, hepatitis C virus status), serum albumin survived false discovery rate (FDR)-correction in contributing variance to larger periventricular but not deep WMH volumes. This relationship was salient in the AUD and HIV groups, but not the control group. In secondary analyses, serum albumin and periventricular WMH along with age, sex, diagnoses, BMI, and hypertension were considered for hierarchical contribution to variance in performance in 4 cognitive domains. Albumin survived FDR-correction for significantly contributing to visual and verbal learning and memory performance after accounting for diagnosis. Relations between albumin and markers of liver integrity [e.g., aspartate transaminase (AST)] and blood status (e.g., hemoglobin, red blood cell count, red cell distribution width) suggest that in this sample, albumin reflects both liver dysfunction and hematological abnormalities. The current results suggest that albumin, a simple serum biomarker available in most clinical settings, can predict variance in periventricular WMH volumes and performance in visual and verbal learning and memory cognitive domains. Whether serum albumin contributes mechanistically to periventricular WMH prevalence will require additional investigation.

## Introduction

Albumin in urine, typically quantified as the urine albumin to creatinine ratio (uACR), is present as a continuous variable ranging from very low in healthy individuals to high fractions (e.g., > 30mg/g microalbuminuria, > 300mg/g macroalbuminuria) in nephrotic syndrome [1, 2]. Albuminuria is independently associated with increased incidence of cardiovascular events after consideration of traditional risk factors such as high body mass index (BMI), nicotine use, diabetes, and hypertension [3–7]. Because albuminuria marks general vascular dysfunction, a 2007 study [8] evaluated the extent to which it would explain incidence of white matter hyperintensities (WMH), considered a neuroimaging feature of cerebral small vessel disease [9]. A community sample of 651 elderly (> 61 years) in Yamagata, Japan was the first to provide evidence that high urine albumin levels (i.e., uACR values > 30 mg/g) are associated with WMH prevalence independent of previously established risk factors (i.e., age, sex, hypertension, diabetes, nicotine use, alcohol consumption) [8]. The relationship between urinary albumin and WMH burden has since been replicated in both healthy and clinical populations [e.g., 1,253 hypertensive participants, Mayo Clinic, MN, US [10]; 285 hypertensive participants, Nagoya, Japan [11]; 975 hypertensive participants, Barcelona, Spain [12]; 1,215 healthy older (> 60 years) adults, Seoul, Korea [13] ; 2,671 healthy older (~ 75 years) adults, Reykjavik, Iceland [14]; 1,214 community dwelling adults, Fukuoka, Japan [15]; 112 at risk elderly (~ 68 years), Exeter, UK [16]; 5,324 patients taking oral antithrombotic agents across multiple sites in Japan [17]].

Low serum albumin levels (i.e., hypoalbuminemia) can occur in several diseases (e.g., kidney disease, liver cirrhosis, malnutrition, malignancy, sepsis) due to mechanisms such as increased excretion, increased or impaired tissue distribution or utilization, increased catabolism, or decreased liver synthesis [18–21]. Other clinical laboratory measures are often used to help determine whether low serum albumin reflects liver disease [e.g., hepatic enzymes aspartate transaminase (AST), alanine transaminase (ALT)], kidney disease [e.g., estimated glomerular filtration rate (eGFR)], malnutrition, or inflammation [e.g., high C-reactive protein (CRP)] [21–24]. Like albuminuria, hypoalbuminemia has been shown to predict cardiovascular incidents (e.g., coronary artery disease, myocardial infarctions, heart failure, arrhythmias) and stroke after consideration of conventional cardiovascular risk factors [25–37]. A major driver of oncotic pressure, vascular functions of serum albumin include binding of fatty acids (i.e., maintaining blood lipid levels) [38] and reactive oxygen species scavenging [39]. Generally, urine and serum albumin levels are inversely related [21, 23, 40–43], but albuminuria does not result in hypoalbuminemia unless total protein loss is in the nephrotic range [44].

Relationships between low serum albumin and cognitive impairment have been described in a variety of clinical populations [e.g., 1,511 patients with heart failure, albumin < 3.5g/dL, multiple sites, Italy [45]; 1,284 adults ≥55 years, albumin = 4.4±0.3g/dL, Amsterdam, Netherlands [46]; 2,550 Chinese adults ≥55 years, albumin < 4.0g/dL, Singapore [47]; 433 hip fracture patients ≥65 years, albumin = 3.51±0.47g/dL, Israel [48]; 1,752 adults ≥65 years, albumin = 3.9±0.4g/dL, multiple sites, England [49]; 191 patients with Parkinson’s Disease, albumin levels unspecified, Pennsylvania, U.S. [50]; 274 patients with acute heart failure, albumin = 3.4±0.4g/dL, Osaka, Japan [51]]. Of note, serum albumin can be low, but not below clinical cutoffs to affect cognitive performance [47–49]. Surprisingly, despite the accruing evidence for the association between albuminuria and WMH prevalence, the correlation between serum albumin and WHM occurrence has rarely been explored. In 46 patients with systemic lupus erythematosus, low serum albumin (i.e., albumin = 3.8±1.3g/dL) was associated with larger WMH lesion volume [52]. Further, CNS edema in posterior reversible encephalopathy syndrome [53–55] and hepatic encephalopathy [56, 57] has been shown to relate to low serum albumin levels. By contrast, a study of 396 older (> 55 years) healthy adults in Korea showed that serum albumin as a continuous variable was associated with high cerebral β amyloid reactivity but not with WMH volume [58].

People infected with the human immunodeficiency virus (HIV) [59–61] or diagnosed with an alcohol use disorder (AUD) [62, 63] demonstrate greater liability for cerebrovascular events than the general population. WMHs are prevalent in HIV and AUD [59, 64–66] and their volume enlarges at an accelerated rate in HIV relative to healthy controls [67]. Both HIV [68, 69] and AUD [70, 71] are associated with lower than control serum albumin and have high rates of hepatitis C virus (HCV) infection comorbidity [72–74], which can independently lower serum albumin [75–80]. Here, cross-sectional data comprising WMH volumes matched to clinical laboratory measures from 160 individuals with AUD, 142 infected with HIV, nd 102 healthy controls were evaluated to test the hypothesis that low serum albumin would predict larger WMH volumes and worse cognitive performance irrespective of diagnosis.

## Methods

### Participants

Cross-sectional neuroimaging and clinical laboratory data from 3 study groups (102 control, 160 AUD, 142 HIV) were extracted from a longitudinal dataset [67] drawn from published studies [67, 81, 82]. Participants were recruited from local alcohol and drug recovery centers, HIV clinics, postcard mailings, recruitment flyers, and word of mouth. After obtaining written informed consent for study participation, approved by the SRI International and Stanford University School of Medicine Institutional Review Boards, volunteers underwent a Structured Clinical Interview for Diagnostic and Statistical Manual (DSM)-IV and DSM-5 Disorders (SCID) [83], structured health questionnaires, and a semi-structured timeline follow-back interview to quantify lifetime alcohol consumption [84]. Upon initial assessment, subjects were excluded if they had a significant history of medical (e.g., epilepsy, stroke, multiple sclerosis, uncontrolled diabetes, or loss of consciousness > 30 minutes), neurological (e.g., Parkinson’s disease), or psychiatric (e.g., schizophrenia, bipolar disorder) disorders other than an AUD (DSM-5). Other exclusionary criteria were substance dependence (other than alcohol for the AUD group) within the past 3 months or any other DSM disorder (for all groups). All participants also completed screening to ensure MRI safety and a breathalyzer test for recent alcohol consumption. Socioeconomic status (SES) was derived from the *Four-Factor Index of Social Status*, which considers education and occupation level and wherein a lower score reflects higher status [85]. As in our other studies, the diagnostic groups relative to the healthy control group were less educated, had worse SES, and were more likely to include men, Black individuals, nicotine use, and hepatitis C virus (HCV) infection (**Table 1**) [81, 82, 86, 87].

### Neuroimaging acquisition and analysis

#### Protocols and parameters.

Scanning was conducted at SRI International on a GE

Discovery MR750 system (Waukesha, WI, U.S.A.) with ASSET for parallel and accelerated imaging on an 8-channel head coil. Detection and localization of WMH used three magnetic resonance imaging (MRI) acquisition protocols: T1-weighted (T1-w) MRI for anatomical localization: 3D axial IR-Prep (inversion prepared) SPGR (SPoiled Gradient Recalled); repetition time (TR) = 6.5ms, echo time (TE) = 1.54ms, thickness (thick) = 1.25 mm, locations (loc) = 124, skip = 0); T2-weighted (T2-w) MRI merged with T1-w data for skull stripping: 3D isotropic FSE (Fast Spin Echo; GE name = CUBE), TR = 2500ms, effective TE = 99ms, echo train length (ETL) = 100ms, thick = 1mm, loc = 150, FOV = 256mm, xy_matrix = 256×256, resolution = 1×1×1mm; and FLAIR (FLuid-Attenuated Inversion Recovery) imaging for estimates of WMH volumes: 2D axial, TR = 9000ms, TE = 82.5ms, inversion time (TI) = 2200ms, thick = 2.5mm, loc = 65.

#### MRI structural analysis.

Preprocessing of T1-weighted SPGR data involved noise removal [88] and brain mask segmentation using FSL BET [89], AFNI 3dSkullStrip [90], and Robust Brain Extraction (ROBEX) [91] generating 3 brain masks. In parallel, noise-corrected, T1-weighted images were corrected for field inhomogeneity via N4ITK [92], brain masks were segmented [93], and the resulting segmented brain masks were reduced to one using majority voting [94]. Brain tissue segmentation (gray matter, white matter, and cerebrospinal fluid) of the skull-stripped T1-weighted images was generated via Atropos [92]. Parcellated maps of tissue used the parc116 atlas to define cortical (gray matter) and subcortical (gray and white matter) volumes summed for bilateral hemispheres.

#### WMH quantification.

WMH analysis was accomplished with the “UBO Detector,” a cluster-based, fully automated pipeline for extracting and calculating WMHs on a voxel basis [95]. This procedure yielded voxel maps for 3 WMH volumes: total, periventricular, and deep. Analysis required that FLAIR and T1-w data be warped into MNI space prior to non-rigid transformation into standard SRI atlas space. This was necessary for accurate placement of anatomical locations to enable comparisons across individuals and across imaging modalities on a voxel-wise basis without the need for further correction for differences in intracranial volume.

### Blood Sample Collection

Blood samples were collected in house for analysis by Quest Diagnostics for complete blood count (CBC) (test: 6399, CPT: 85025), comprehensive metabolic panel (test code 10231, CPT code 80053), and HIV and HCV screening with RNA quantification for seropositive individuals. CBC required whole blood collected in EDTA tubes; remaining tests used serum separator tubes (SST) tubes. The Quest Diagnostics reference range for albumin is 3.6–5.1g/dL; levels < 3.5g/dL were considered out of range [96].

To evaluate the significance of albumin, its relations with other blood biomarkers were considered. For liver disease, relations between albumin and levels of AST, ALT, *γ*-glutamyl transferase (GGT), alkaline phosphatase, and prealbumin were evaluated; for kidney disease, eGFR and creatinine levels; for malnutrition, levels of vitamins B9 (folate) and B12 (cobalamin); and for inflammation TNFα and IP10 levels [for details on methods for cytokine measures see: [86, 97]]

### Cognitive Composite Scores

Cognitive composite scores matched to date of blood draw for each participant were extracted from an in-house laboratory release as described [67, 98]. Briefly, composites cognitive scores were created by averaging age-, education-, and sex-corrected Z-scores on performance on neuropsychological tests. Composites scores comprised tests of executive functioning, attention and working memory, visual and verbal learning, and visual and verbal memory as listed.

#### executive functioning

Trails B time *or* Color-trails time 2 +

Digit symbol raw score at 90 sec or Symbol digit raw score at 90 sec +

Phonological fluency (sum of unique “F” + “A” + “S” words)

#### attention and working memory

Trails A time *or* Color-trails time1 +

Wechsler Memory Scale-Revised (WMS-R) digits forward raw score total +

WMS-R digits backwards raw score total +

WMS-R block tapping forward total +

WMS-R block tapping backward total

#### visual and verbal learning

Rey-Osterrieth complex figure immediate raw score +

WMS-R logical memory immediate total

#### visual and verbal memory

Rey-Osterrieth complex figure delay raw score +

WMS-R logical memory delay total

### Statistics

Statistics were performed using JMP^®^ Pro 16.0.0 (SAS Institute Inc., Cary, NC, 1989–2021). For comparisons, χ^2^ was used on categorical variables and Welch’s test of unequal variances was used for continuous variables. Correlations were evaluated using simple linear regressions. Significance required Bonferroni-corrected p-values as indicated in tables. Initial stepwise regression models to predict 3 WMH volumes from 11 variables [age, sex (male/female), race (black/white/other), BMI, SES, diagnosis (controls/AUD/HIV/AUD + HIV), HCV status (positive/negative), nicotine use (never/past or current), hypertension (yes/no, yes = systolic≥140 or diastolic≥90), diabetes (yes/no, self-report), and albumin levels] were followed by multiple regression analyses including only the variables indicated by the stepwise regression. Prediction of performance in 4 cognitive domains used multiple regressions considering variables relevant to WMH volumes. Significant variables identified by multiple regressions list the FDR (false discovery rate)-corrected logworth contribution (defined as −log_10_[p-value], which adjusts p-values to provide a standardized scale) to total variance.

## Results

**Figure 1:**
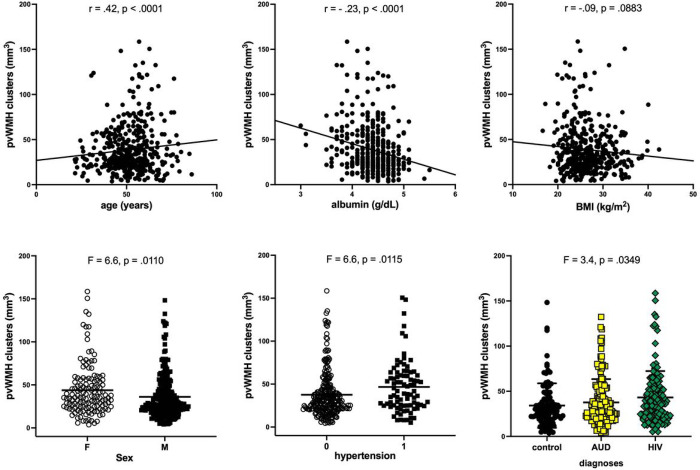
Variables that met false discovery rate (FDR)-corrected significance in contributing to periventricular WMH volumes (i.e., pvWMH clusters) including age, albumin, body mass index (BMI), sex, hypertension (i.e., systolic≥140 or diastolic≥190), and diagnoses (control, AUD, HIV).

Albumin levels were lower in the HIV relative to the AUD and healthy control groups; only 6 HIV participants, however, had serum albumin levels < 3.5g/dL. For each WMH volume (i.e., periventricular, deep, total), a stepwise regression considered 11 factors: age, sex, race, SES, BMI, diagnosis, HCV status, nicotine use, presence of hypertension or diabetes, and albumin levels. For periventricular WMH volume, the stepwise regression selected 6/11 variables: age, sex, BMI, diagnoses, hypertension, and albumin levels. The follow-up multiple regression model to predict periventricular WMH volume was significant (F7,363=17.8, p < .0001), explained 25.9% of the variance (i.e., R^2^), and all 6 variables passed FDR significance for contribution to periventricular WMH volume driven by age (−log_10_[FDR p-value] = 14.2), with similar contributions from albumin (−log_10_[FDR p-value] = 2.1), BMI (−log_10_[FDR p-value] = 2.1), sex (−log10[FDR p-value] = 2.1), and diagnoses (−log10[FDR p-value] = 2.1); then hypertension (−log10[FDR p-value] = 1.9) (Fig. 1). The AUD (r=−.25, p = .0016) and HIV (r=−.23, p = .0066) groups but not the control group (r=−.08, p = .4391) showed an inverse relationship between albumin and periventricular WMH volume. If the control group is removed from the analyses, the multiple regression to predict periventricular WMH volume from the 6 variables remains significant (F6,260=12.2, p < .0001), explains 22.4% of the variance and is driven by age (−log_10_[FDR p-value] = 7.9), then BMI (−log_10_[FDR p-value] = 2.3), then equal contributions from albumin and sex (−log_10_[FDR p-value] = 2.2); in this model, hypertension and diagnoses do not survive FDR correction.

For total WMH volume, the stepwise regression selected the same 6 variables as those chosen for periventricular WMH. The multiple regression model was significant (F7,363=20.4, p < .0001), explained 28.6% of the variance, and was driven by age (−log_10_[FDR p-value] = 17.8); diagnoses (−log_10_[FDR p-value] = 2.0) and BMI (−log_10_[FDR p-value] = 2.0) contributed next; then sex (−log_10_[FDR p-value] = 1.8), albumin (−log10[FDR p-value] = 1.8), and hypertension (−log10[FDR p-value] = 1.7).

For deep WMH volume, the stepwise regression selected 5/11 variables: age, BMI, diagnoses, hypertension, and albumin (i.e., same as those selected for periventricular WMH minus sex). The follow-up multiple regression model to predict deep WMH volume explained 25.1% of the variance (F6,363=20.0, p < .0001) and was driven by age (−log_10_[FDR p-value] = 18.0); none of the other variables contributed significantly with FDR-corrected significance to deep WMH volumes.

**Figure 2:**
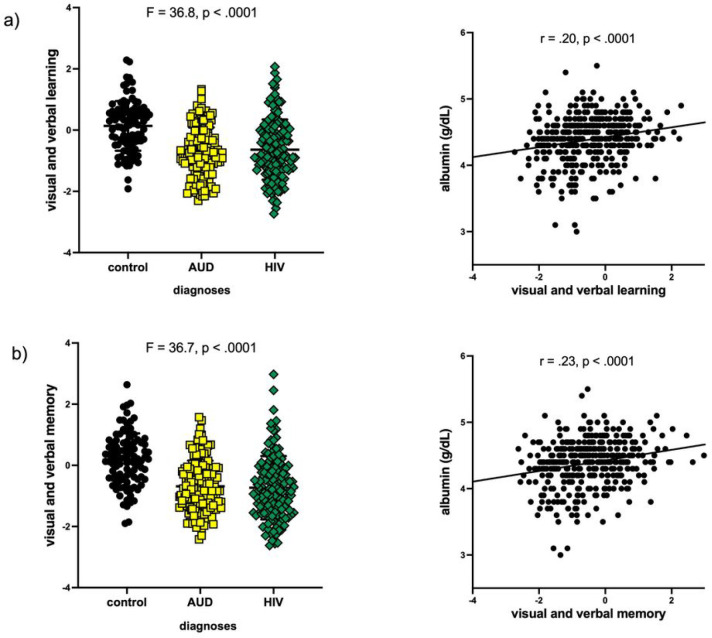
a) performance by diagnostic group on visual and verbal learning (top) or memory (bottom); b) relations between albumin and visual and verbal learning (top) or memory (bottom) cognitive composite scores.

Next considered were the contributions of albumin levels and periventricular WMH volumes to cognitive performance in 4 domains. For these 4 multiple regressions (i.e., to predict performance on executive, attention and working memory, visual and verbal learning, or visual and verbal memory), a total of 7 variables were considered (i.e., age, sex, BMI, hypertension, diagnoses, albumin, periventricular WMH volume). All 4 models were significant (p < .0001) and explained 15.0–20.6% of the variance in cognitive performance (**Table S1**). Albumin levels after diagnoses survived contributing FDR-corrected significance to visual and verbal learning [F8,349=10.8, p < .0001, R^2^ = 20.2; diagnoses (−log10[FDR p-value] = 10.9), albumin (−log10[FDR p-value] = 2.6)] and visual and verbal memory [F8,349=11.0, p < .0001, R^2^ = 20.6; diagnoses (−log_10_ [FDR p-value] = 10.4), albumin (−log_10_[FDR p-value] = 2.9)] (Fig. 2); the other 5 variables did not contribute to performance on these 2 composite cognitive scores.

Finally, to evaluate whether low albumin levels reflect malnutrition, inflammation, liver, kidney, or cardiovascular disease, relations between albumin or HCV and other blood markers were considered (**Table 2**). Both albumin and HCV correlated with markers of liver integrity (e.g., AST, prealbumin); only albumin was additionally associated with hematological markers (e.g., red blood cell count, red cell distribution width).

## Discussion

As has been demonstrated for high urine albumin [8, 11–17], the current results suggest that low serum albumin, even at levels above clinical cutoffs, explains some of the variance in WMH volumes. Here, lower serum albumin in a cohort comprising individuals with AUD and HIV was associated with larger periventricular WMH volumes. Further, albumin but not periventricular WMH contributed to performance on visual and verbal learning and memory. Finally, relations between albumin and other common blood markers suggest that low albumin reflects liver disease and hematological abnormalities.

This study was motivated by the consistent observation since 2007 that high urine albumin levels predict WMH prevalence in both healthy and clinical populations [8, 11–17]. As both albuminuria and hypoalbuminemia predict cardiovascular event prevalence independent of traditional risk factors, however, it is unexpected that the serum marker has not been more widely evaluated for its contribution to WMH volume. Only 2 previous reports interrogating the relationship between serum albumin and WMH prevalence have been published with the assessment in those with systemic lupus erythematosus demonstrating an inverse relationship [52] while that in healthy older adults did not [58]. A study using albumin relative to globulin, however, did identify this ratio as relevant to predicting the WMH severity among cerebrovascular risk patients [99]. Our results comport with these previous studies in suggesting that, despite levels in the “normal range”, low serum albumin may predict WMH volume in clinical populations (i.e., AUD, HIV).

The current results also confirm and extend the literature demonstrating relations between low serum albumin and cognitive impairment [e.g., 45, 48, 49]. For example, in patients with acute heart failure, those with cognitive impairment (albumin = 3.2±0.5g/dL) as determined using the Mini Mental State Examination (MMSE) relative to those with normal cognitive functioning (albumin = 3.6±0.5g/dL) had significantly lower albumin levels [51]. Similarly, in elderly patients with epilepsy, those with MMSE-determined cognitive impairment relative to those without impairment showed lower albumin [100]. Other clinical populations also demonstrate a relation between cognitive impairment and low serum albumin [e.g., Parkinson’s disease [50], post-stroke patients with type 2 diabetes mellitus [101], older adults with cognitive impairment [102, 103], elderly dialysis patients [104–106]]. Indeed, HIV mono-infected [107] and HIV + HCV co-infected [108] individuals with cognitive impairment, including disturbed visual memory in HIV + HCV-coinfection [109], have low serum albumin. Similarly, an association between cognitive dysfunction determined using the cognitive failures questionnaire and low albumin was described in alcohol-related liver disease [110].

Differences in blood marker correlates between HCV and albumin suggest that albumin functions more than just an indicator of liver status. That is, whereas both HCV and albumin correlated with serum markers of liver function (i.e., aspartate transaminase, prealbumin; note, although prealbumin is sometimes considered a nutritional marker, Quest Diagnostics, states that “prealbumin is decreased in liver disease”), only albumin additionally correlated with hematological markers (i.e., lower red blood cell count, lower hemoglobin, lower hematocrit, and higher red cell distribution width). These relations between albumin and altered hemodynamic profiles have previously been reported [111] and may reflect cardiovascular dysfunction [112–117].

In conclusion, the current study contributes to a nascent literature demonstrating relations between serum albumin and WMH burden in clinical populations including those with AUD and HIV. Whether low serum albumin contributes mechanistically to periventricular WMH will require additional investigation. Despite evidence that low serum albumin is a reliable biomarker of vascular endothelial function and can cause edema [118], it is unclear whether it contributes to hypertension [119] or affects blood flow [120]. However, evidence that hypertension [121] or a decline in total cerebral blood flow [122] can increase periventricular but not deep WMH volumes invites the speculation that albumin may contribute to increasing periventricular WMH prevalence via effects on hypertension or altering blood flow.

## Data Availability

Data described in the manuscript, code book, and analytic code will be made publicly and freely available without restriction at https://data.mendeley.com/.

## References

[1] Vestergaard SV, Birn H, Hansen AT, Nørgaard M, Nitsch D, Christiansen CF. Comparison of patients with hospital-recorded nephrotic syndrome and patients with nephrotic proteinuria and hypoalbuminemia: A nationwide study in denmark. Kidney360 2021;2:1482–90. doi: 10.34067/kid.0000362021 35373110 PMC8786138

[2] Becker GJ. Which albumin should we measure? Kidney Int Suppl 2004:S16–7. doi: 10.1111/j.1523-1755.2004.09204.x 15485408

[3] Jensen JS, Feldt-Rasmussen B, Strandgaard S, Schroll M, Borch-Johnsen K. Arterial hypertension, microalbuminuria, and risk of ischemic heart disease. Hypertension 2000;35:898–903. doi: 10.1161/01.hyp.35.4.898 10775558

[4] Cao JJ, Barzilay JI, Peterson D, Manolio TA, Psaty BM, Kuller L, The association of microalbuminuria with clinical cardiovascular disease and subclinical atherosclerosis in the elderly: The cardiovascular health study. Atherosclerosis 2006;187:372–7. doi: 10.1016/j.atherosclerosis.2005.09.015 16242696

[5] Nakayama T, Date C, Yokoyama T, Yoshiike N, Yamaguchi M, Tanaka H. A 15.5-year follow-up study of stroke in a japanese provincial city. The shibata study. Stroke 1997;28:45–52. doi: 10.1161/01.str.28.1.45 8996487

[6] Beamer NB, Coull BM, Clark WM, Wynn M. Microalbuminuria in ischemic stroke. Arch Neurol 1999;56:699–702. doi: 10.1001/archneur.56.6.699 10369309

[7] Ravera M, Ratto E, Vettoretti S, Viazzi F, Leoncini G, Parodi D, Microalbuminuria and subclinical cerebrovascular damage in essential hypertension. J Nephrol 2002;15:519–24. 12455718

[8] Wada M, Nagasawa H, Kurita K, Koyama S, Arawaka S, Kawanami T, Microalbuminuria is a risk factor for cerebral small vessel disease in community-based elderly subjects. J Neurol Sci 2007;255:27–34. doi: 10.1016/j.jns.2007.01.066 17320908

[9] Wardlaw JM, Smith EE, Biessels GJ, Cordonnier C, Fazekas F, Frayne R, Neuroimaging standards for research into small vessel disease and its contribution to ageing and neurodegeneration. Lancet Neurol 2013;12:822–38. doi: 10.1016/s1474-4422(13)70124-8 23867200 PMC3714437

[10] Knopman DS, Mosley TH, Jr., Bailey KR, Jack CR, Jr., Schwartz GL, Turner ST. Associations of microalbuminuria with brain atrophy and white matter hyperintensities in hypertensive sibships. J Neurol Sci 2008;271:53–60. doi: 10.1016/j.jns.2008.03.009 18442832 PMC2527625

[11] Umemura T, Kawamura T, Sakakibara T, Mashita S, Hotta N, Sobue G. Microalbuminuria is independently associated with deep or infratentorial brain microbleeds in hypertensive adults. Am J Hypertens 2012;25:430–6. doi: 10.1038/ajh.2011.254 22237153

[12] Vilar-Bergua A, Riba-Llena I, Ramos N, Mundet X, Espinel E, López-Rueda A, Microalbuminuria and the combination of mri markers of cerebral small vessel disease. Cerebrovasc Dis 2016;42:66–72. doi: 10.1159/000445168 27031692

[13] Cho EB, Shin HY, Park SE, Chun P, Jang HR, Yang JJ, Albuminuria, cerebrovascular disease and cortical atrophy: Among cognitively normal elderly individuals. Sci Rep 2016;6:20692. doi: 10.1038/srep20692 26878913 PMC4754729

[14] Sedaghat S, Ding J, Eiriksdottir G, van Buchem MA, Sigurdsson S, Ikram MA, The ages-reykjavik study suggests that change in kidney measures is associated with subclinical brain pathology in older community-dwelling persons. Kidney Int 2018;94:608–15. doi: 10.1016/j.kint.2018.04.022 29960746 PMC6190704

[15] Yamasaki K, Hata J, Furuta Y, Hirabayashi N, Ohara T, Yoshida D, Association of albuminuria with white matter hyperintensities volume on brain magnetic resonance imaging in elderly Japanese - the hisayama study. Circ J 2020;84:935–42. doi: 10.1253/circj.CJ-19-1069 32269184

[16] Elyas S, Adingupu D, Aizawa K, Casanova F, Gooding K, Fulford J, Cerebral small vessel disease, systemic vascular characteristics and potential therapeutic targets. Aging (Albany NY) 2021;13:22030–9. doi: 10.18632/aging.203557 34550097 PMC8507297

[17] Tanaka K, Miwa K, Takagi M, Sasaki M, Yakushiji Y, Kudo K, Increased cerebral small vessel disease burden with renal dysfunction and albuminuria in patients taking antithrombotic agents: The bleeding with antithrombotic therapy 2. J Am Heart Assoc 2022;11:e024749. doi: 10.1161/jaha.121.024749 35253443 PMC9075282

[18] Fleck A, Hawker F, Wallace P, Raines G, Trotter J, Ledingham IM, Increased vascular permeability: A major cause of hypoalbuminaemia in disease and injury. The Lancet 1985;325:781–4. 10.1016/s0140-6736(85)91447-3 2858667

[19] Barchel D, Almoznino-Sarafian D, Shteinshnaider M, Tzur I, Cohen N, Gorelik O. Clinical characteristics and prognostic significance of serum albumin changes in an internal medicine ward. Eur J Intern Med 2013;24:772–8. doi: 10.1016/j.ejim.2013.08.004 24011640

[20] Gatta A, Verardo A, Bolognesi M. Hypoalbuminemia. Intern Emerg Med 2012;7 Suppl 3: S193–9. doi: 10.1007/s11739-012-0802-0 23073857

[21] Gounden V, Vashisht R, Jialal I. Hypoalbuminemia. In: Treasure Island (FL): StatPearls Publishing; 2023. 30252336

[22] Bharadwaj S, Ginoya S, Tandon P, Gohel TD, Guirguis J, Vallabh H, Malnutrition: Laboratory markers vs nutritional assessment. Gastroenterol Rep (Oxf) 2016;4:272–80. doi: 10.1093/gastro/gow013 27174435 PMC5193064

[23] Caironi P, Gattinoni L. The clinical use of albumin: The point of view of a specialist in intensive care. Blood Transfus 2009;7:259–67. doi: 10.2450/2009.0002-09 20011637 PMC2782803

[24] World Health Organization. Second who model list of essential in vitro diagnostics. . Geneva: World Health Organization (WHO/MVP/EMP/2019.05). 2019.

[25] Phillips A, Shaper AG, Whincup P. Association between serum albumin and mortality from cardiovascular disease, cancer, and other causes. The Lancet 1989;334:1434–6. 10.1016/s0140-6736(89)92042-4 2574367

[26] Gillum RF, Ingram DD, Makuc DM. Relation between serum albumin concentration and stroke incidence and death: The nhanes i epidemiologic follow-up study. Am J Epidemiol 1994;140:876–88. doi: 10.1093/oxfordjournals.aje.a117176 7977275

[27] Goldwasser P, Feldman J. Association of serum albumin and mortality risk. J Clin Epidemiol 1997;50:693–703. doi: 10.1016/s0895-4356(97)00015-2 9250267

[28] Enia G, Mallamaci F, Benedetto FA, Panuccio V, Parlongo S, Cutrupi S, Long-term capd patients are volume expanded and display more severe left ventricular hypertrophy than haemodialysis patients. Nephrology Dialysis Transplantation 2001;16:1459–64. doi: 10.1093/ndt/16.7.1459 11427641

[29] Feldman JG, Gange SJ, Bacchetti P, Cohen M, Young M, Squires KE, Serum albumin is a powerful predictor of survival among hiv-1-infected women. J Acquir Immune Defic Syndr 2003;33:66–73. doi: 10.1097/00126334-200305010-00010 12792357

[30] Høstmark AT, Tomten SE. Serum albumin and self-reported prevalence of stroke: A population-based, cross-sectional study. Eur J Cardiovasc Prev Rehabil 2006;13:87–90. doi: 10.1097/00149831-200602000-00013 16449869

[31] Lang J, Scherzer R, Weekley CC, Tien PC, Grunfeld C, Shlipak MG. Serum albumin and short-term risk for mortality and cardiovascular disease among hiv-infected veterans. Aids 2013;27:1339–43. doi: 10.1097/QAD.0b013e32835f1dd6 23343914 PMC4026018

[32] Xu W-H, Dong C, Rundek T, Elkind MS, Sacco RL. Serum albumin levels are associated with cardioembolic and cryptogenic ischemic strokes: Northern manhattan study. Stroke 2014;45:973–8. 24549868 10.1161/STROKEAHA.113.003835 PMC3966953

[33] Ancion A, Allepaerts S, Robinet S, Oury C, Pierard LA, Lancellotti P. Serum albumin level and long-term outcome in acute heart failure. Acta Cardiologica 2019;74:465–71. doi: 10.1080/00015385.2018.1521557 30650026

[34] Prenner SB, Kumar A, Zhao L, Cvijic ME, Basso M, Spires T, Effect of serum albumin levels in patients with heart failure with preserved ejection fraction (from the topcat trial). The American Journal of Cardiology 2020;125:575–82. doi: 10.1016/j.amjcard.2019.11.006 31843232 PMC6986988

[35] Arques S. Serum albumin and cardiovascular disease: State-of-the-art review. Annales de Cardiologie et d’Angéiologie. Elsevier; 2020, p. 192–200. 10.1016/j.ancard.2020.07.012 32797938

[36] Li X, Zhang Y, He Y, Li KX, Xu RN, Wang H, J-shaped association between serum albumin levels and long-term mortality of cardiovascular disease: Experience in national health and nutrition examination survey (2011–2014). Front Cardiovasc Med 2022;9:1073120. doi: 10.3389/fcvm.2022.1073120 36523355 PMC9745145

[37] Zhao D, Jiao H, Zhong X, Wang W, Li L. The association between serum albumin levels and related metabolic factors and atrial fibrillation: A retrospective study. Medicine (Baltimore) 2022;101:e31581. doi: 10.1097/md.0000000000031581 36343084 PMC9646583

[38] Peters T, Jr. Serum albumin. Adv Protein Chem 1985;37:161–245. doi: 10.1016/s0065-3233(08)60065-0 3904348

[39] Iglesias J, Abernethy VE, Wang Z, Lieberthal W, Koh JS, Levine JS. Albumin is a major serum survival factor for renal tubular cells and macrophages through scavenging of ros. Am J Physiol 1999;277:F711–22. doi: 10.1152/ajprenal.1999.277.5.F711 10564234

[40] Anavekar NS, Gans DJ, Berl T, Rohde RD, Cooper W, Bhaumik A, Predictors of cardiovascular events in patients with type 2 diabetic nephropathy and hypertension: A case for albuminuria. Kidney Int Suppl 2004:S50–5. doi: 10.1111/j.1523-1755.2004.09213.x 15485418

[41] Wang Z, Hoy WE, Wang Z. The correlates of urinary albumin to creatinine ratio (acr) in a high risk australian aboriginal community. BMC Nephrology 2013;14:176. doi: 10.1186/1471-2369-14-176 23947772 PMC3765271

[42] Boorsma EM, ter Maaten JM, Damman K, van Essen BJ, Zannad F, van Veldhuisen DJ, Albuminuria as a marker of systemic congestion in patients with heart failure. European Heart Journal 2022;44:368–80. doi: 10.1093/eurheartj/ehac528 PMC9890244 36148485

[43] Nour M, hegazy A, mosbah A, Abdelaziz A, Fawzy M. Role of microalbuminuria and hypoalbuminemia as outcome predictors in critically ill patients. Critical Care Research and Practice 2021;2021:6670642. doi: 10.1155/2021/6670642 33953981 PMC8057906

[44] Levitt DG, Levitt MD. Human serum albumin homeostasis: A new look at the roles of synthesis, catabolism, renal and gastrointestinal excretion, and the clinical value of serum albumin measurements. Int J Gen Med 2016;9:229–55. doi: 10.2147/ijgm.S102819 27486341 PMC4956071

[45] Zuccalà G, Marzetti E, Cesari M, Monaco MRL, Antonica L, Cocchi A, Correlates of cognitive impairment among patients with heart failure: Results of a multicenter survey. The American journal of medicine 2005;118:496–502. 15866252 10.1016/j.amjmed.2005.01.030

[46] Dik MG, Jonker C, Hack CE, Smit JH, Comijs HC, Eikelenboom P. Serum inflammatory proteins and cognitive decline in older persons. Neurology 2005;64:1371–7. doi: 10.1212/01.Wnl.0000158281.08946.68 15851726

[47] Ng TP, Feng L, Niti M, Yap KB. Albumin, haemoglobin, bmi and cognitive performance in older adults. Age Ageing 2008;37:423–9. doi: 10.1093/ageing/afn102 18495687

[48] Mizrahi EH, Blumstein T, Arad M, Adunsky A. Serum albumin levels predict cognitive impairment in elderly hip fracture patients. American Journal of Alzheimer’s Disease & Other Dementias^®^ 2008;23:85–90. 10.1177/1533317507311776 PMC10846191 18174316

[49] Llewellyn DJ, Langa KM, Friedland RP, Lang IA. Serum albumin concentration and cognitive impairment. Current Alzheimer Research 2010;7:91–6. 20205675 10.2174/156720510790274392 PMC2886725

[50] Shen J, Amari N, Zack R, Skrinak RT, Unger TL, Posavi M, Plasma mia, crp, and albumin predict cognitive decline in parkinson’s disease. Ann Neurol 2022;92:255–69. doi: 10.1002/ana.26410 35593028 PMC9329215

[51] Seo M, Watanabe T, Yamada T, Morita T, Kawasaki M, Kikuchi A, The clinical relevance of mild cognitive impairment in acute heart failure: A comparison with cognitive impairment. J Cardiol 2023. doi: 10.1016/j.jjcc.2023.08.017 37684004

[52] Shaharir SS, Osman SS, Md Rani SA, Sakthiswary R, Said MSM. Factors associated with increased white matter hyperintense lesion (wmhi) load in patients with systemic lupus erythematosus (sle). Lupus 2018;27:25–32. doi: 10.1177/0961203317707062 28467290

[53] Gao B, Yu BX, Li RS, Zhang G, Xie HZ, Liu FL, Cytotoxic edema in posterior reversible encephalopathy syndrome: Correlation of mri features with serum albumin levels. American Journal of Neuroradiology 2015;36:1884–9. doi: 10.3174/ajnr.A4379 26138140 PMC7965038

[54] Pirker A, Kramer L, Voller B, Loader B, Auff E, Prayer D. Type of edema in posterior reversible encephalopathy syndrome depends on serum albumin levels: An mr imaging study in 28 patients. American journal of neuroradiology 2011;32:527–31. 21252042 10.3174/ajnr.A2332 PMC8013074

[55] Fang XB, Chen DJ, He F, Chen J, Zhou Z, Liang YL, Predictors of oedema type in reversible posterior leukoencephalopathy syndrome with preeclampsia or eclampsia. Pregnancy Hypertens 2018;11:71–6. doi: 10.1016/j.preghy.2017.12.011 29523278

[56] Tran TT, Wei K, Cole S, Mena E, Csete M, King KS. Brain mr spectroscopy markers of encephalopathy due to nonalcoholic steatohepatitis. J Neuroimaging 2020;30:697–703. doi: 10.1111/jon.12728 32705733

[57] Solomou E, Velissaris D, Polychronopoulos P, Kalogeropoulos A, Kiriakopoulou M, Mpadra F, Quantitative evaluation of magnetic resonance imaging (mri) abnormalities in subclinical hepatic encephalopathy. Hepatogastroenterology 2005;52:203–7. 15783031

[58] Kim JW, Byun MS, Lee JH, Yi D, Jeon SY, Sohn BK, Serum albumin and beta-amyloid deposition in the human brain. Neurology 2020;95:e815–e26. doi: 10.1212/wnl.0000000000010005 32690787 PMC7605506

[59] Stewart CR, Stringer MS, Shi Y, Thrippleton MJ, Wardlaw JM. Associations between white matter hyperintensity burden, cerebral blood flow and transit time in small vessel disease: An updated meta-analysis. Front Neurol 2021;12:647848. doi: 10.3389/fneur.2021.647848 34017302 PMC8129542

[60] Murray KD, Uddin MN, Tivarus ME, Sahin B, Wang HZ, Singh MV, Increased risk for cerebral small vessel disease is associated with quantitative susceptibility mapping in hiv infected and uninfected individuals. Neuroimage Clin 2021;32:102786. doi: 10.1016/j.nicl.2021.102786 34500428 PMC8429957

[61] Haddow LJ, Sudre CH, Sokolska M, Gilson RC, Williams IG, Golay X, Magnetic resonance imaging of cerebral small vessel disease in men living with hiv and hiv-negative men aged 50 and above. AIDS Res Hum Retroviruses 2019;35:453–60. doi: 10.1089/aid.2018.0249 30667282 PMC6508064

[62] Lewis JP, Suchy-Dicey AM, Noonan C, Blue Bird Jernigan V, Umans JG, Domoto-Reilly K, Associations of binge drinking with vascular brain injury and atrophy in older american indians: The strong heart study. Journal of aging and health 2021;33:51S–9S. 34167344 10.1177/08982643211013696 PMC8845484

[63] Schutte R, Smith L, Wannamethee G. Alcohol – the myth of cardiovascular protection. Clinical Nutrition 2022;41:348–55. doi: 10.1016/j.clnu.2021.12.009 34999329

[64] Watson C, Busovaca E, Foley JM, Allen IE, Schwarz CG, Jahanshad N, White matter hyperintensities correlate to cognition and fiber tract integrity in older adults with hiv. J Neurovirol 2017;23:422–9. doi: 10.1007/s13365-016-0509-5 28101804 PMC5826904

[65] Seider TR, Gongvatana A, Woods AJ, Chen H, Porges EC, Cummings T, Age exacerbates hiv-associated white matter abnormalities. J Neurovirol 2016;22:201–12. doi: 10.1007/s13365-015-0386-3 26446690 PMC4783252

[66] Samboju V, Cobigo Y, Paul R, Naasan G, Hillis M, Tsuei T, Cerebrovascular disease correlates with longitudinal brain atrophy in virally suppressed older people living with hiv. J Acquir Immune Defic Syndr 2021;87:1079–85. doi: 10.1097/qai.0000000000002683 34153014 PMC8547347

[67] Pfefferbaum A, Zhao Q, Pohl KM, Sassoon SA, Zahr NM, Sullivan EV. Age-accelerated increase of white matter hyperintensity volumes is exacerbated by heavy alcohol use in people living with hiv. Biol Psychiatry 2023. doi: 10.1016/j.biopsych.2023.07.023 PMC10840832 37597798

[68] Mehta SH, Astemborski J, Sterling TR, Thomas DL, Vlahov D. Serum albumin as a prognostic indicator for hiv disease progression. AIDS Research and Human Retroviruses 2006;22:14–21. doi: 10.1089/aid.2006.22.14 16438640

[69] Ronit A, Sharma S, Baker JV, Mngqibisa R, Delory T, Caldeira L, Serum albumin as a prognostic marker for serious non-aids endpoints in the strategic timing of antiretroviral treatment (start) study. J Infect Dis 2018;217:405–12. doi: 10.1093/infdis/jix350 29244111 PMC5853310

[70] Sullivan EV, Pfefferbaum A. Magnetic resonance relaxometry reveals central pontine abnormalities in clinically asymptomatic alcoholic men. Alcohol Clin Exp Res 2001;25:1206–12. 11505052

[71] Torruellas C, French SW, Medici V. Diagnosis of alcoholic liver disease. World J Gastroenterol 2014;20:11684–99. doi: 10.3748/wjg.v20.i33.11684 25206273 PMC4155359

[72] Fuster D, Sanvisens A, Bolao F, Rivas I, Tor J, Muga R. Alcohol use disorder and its impact on chronic hepatitis c virus and human immunodeficiency virus infections. World J Hepatol 2016;8:1295–308. doi: 10.4254/wjh.v8.i31.1295 27872681 PMC5099582

[73] Lieber CS. Alcohol and hepatitis c. Alcohol Res Health 2001;25:245–54. 11910701 PMC6705702

[74] Kim AY, Onofrey S, Church DR. An epidemiologic update on hepatitis c infection in persons living with or at risk of hiv infection. J Infect Dis 2013;207 Suppl 1:S1–6. doi: 10.1093/infdis/jis927 23390299 PMC3565593

[75] Chitturi S, George J. Predictors of liver-related complications in patients with chronic hepatitis c. Ann Med 2000;32:588–91. doi: 10.3109/07853890009002028 11209965

[76] Tillmann HL, Manns MP, Rudolph KL. Merging models of hepatitis c virus pathogenesis. Semin Liver Dis 2005;25:84–92. doi: 10.1055/s-2005-864784 15732000

[77] Toshikuni N, Izumi A, Nishino K, Inada N, Sakanoue R, Yamato R, Comparison of outcomes between patients with alcoholic cirrhosis and those with hepatitis c virus-related cirrhosis. J Gastroenterol Hepatol 2009;24:1276–83. doi: 10.1111/j.1440-1746.2009.05851.x 19486451

[78] Schley G, Köberle C, Manuilova E, Rutz S, Forster C, Weyand M, Comparison of plasma and urine biomarker performance in acute kidney injury. PLOS ONE 2015;10:e0145042. doi: 10.1371/journal.pone.0145042 26669323 PMC4682932

[79] Colombo M, McGurnaghan SJ, Blackbourn LAK, Dalton RN, Dunger D, Bell S, Comparison of serum and urinary biomarker panels with albumin/creatinine ratio in the prediction of renal function decline in type 1 diabetes. Diabetologia 2020;63:788–98. doi: 10.1007/s00125-019-05081-8 31915892 PMC7054370

[80] Krolewski AS. Progressive renal decline: The new paradigm of diabetic nephropathy in type 1 diabetes. Diabetes Care 2015;38:954–62. doi: 10.2337/dc15-0184 25998286 PMC4439536

[81] Pfefferbaum A, Zahr NM, Sassoon SA, Kwon D, Pohl KM, Sullivan EV. Accelerated and premature aging characterizing regional cortical volume loss in human immunodeficiency virus infection: Contributions from alcohol, substance use, and hepatitis c coinfection. Biol Psychiatry Cogn Neurosci Neuroimaging 2018;3:844–59. doi: 10.1016/j.bpsc.2018.06.006 30093343 PMC6508083

[82] Sullivan EV, Zahr NM, Sassoon SA, Thompson WK, Kwon D, Pohl KM, The role of aging, drug dependence, and hepatitis c comorbidity in alcoholism cortical compromise. JAMA Psychiatry 2018. doi: 10.1001/jamapsychiatry.2018.0021 PMC5875381 29541774

[83] First MB, Spitzer RL, Gibbon M, Williams JBW. Structured clinical interview for dsm-iv axis i disorders (scid) version 2.0. New York, NY.: Biometrics Research Department, New York State Psychiatric Institute; 1998.

[84] Skinner HA, Sheu WJ. Reliability of alcohol use indices. The lifetime drinking history and the mast. J Stud Alcohol 1982;43:1157–70. 7182675 10.15288/jsa.1982.43.1157

[85] Hollingshead A. Four-factor index of social status. New Haven, CT: Department of Sociology, Yale University; 1975.

[86] Zahr NM, Pohl KM, Kwong AJ, Sullivan EV, Pfefferbaum A. Preliminary evidence for a relationship between elevated plasma tnfα and smaller subcortical white matter volume in hcv infection irrespective of hiv or aud comorbidity. International Journal of Molecular Sciences 2021;22. doi: 10.3390/ijms22094953 34067023 PMC8124321

[87] Kwong AJ, Zahr NM. Serum biomarkers of liver fibrosis identify globus pallidus vulnerability. NeuroImage: Clinical 2023;37:103333. doi: 10.1016/j.nicl.2023.103333 PMC9996367 36868044

[88] Coupe P, Yger P, Prima S, Hellier P, Kervrann C, Barillot C. An optimized blockwise nonlocal means denoising filter for 3-d magnetic resonance images. IEEE Trans Med Imaging 2008;27:425–41. doi: 10.1109/TMI.2007.906087 18390341 PMC2881565

[89] Smith SM. Fast robust automated brain extraction. Hum Brain Mapp 2002;17:143–55. doi: 10.1002/hbm.10062 12391568 PMC6871816

[90] Cox RW. Afni: Software for analysis and visualization of functional magnetic resonance neuroimages. Comput Biomed Res 1996;29:162–73. 8812068 10.1006/cbmr.1996.0014

[91] Iglesias JE, Liu CY, Thompson PM, Tu Z. Robust brain extraction across datasets and comparison with publicly available methods. IEEE Trans Med Imaging 2011;30:1617–34. doi: 10.1109/TMI.2011.2138152 21880566

[92] Avants BB, Tustison NJ, Song G, Cook PA, Klein A, Gee JC. A reproducible evaluation of ants similarity metric performance in brain image registration. Neuroimage 2011;54:2033–44. doi: 10.1016/j.neuroimage.2010.09.025 20851191 PMC3065962

[93] Sadananthan SA, Zheng W, Chee MW, Zagorodnov V. Skull stripping using graph cuts. Neuroimage 2010;49:225–39. doi: 10.1016/j.neuroimage.2009.08.050 19732839

[94] Rohlfing T, Brandt R, Menzel R, Maurer CR Jr. Evaluation of atlas selection strategies for atlas-based image segmentation with application to confocal microscopy images of bee brains. Neuroimage 2004;21:1428–42. 15050568 10.1016/j.neuroimage.2003.11.010

[95] Jiang J, Paradise M, Liu T, Armstrong NJ, Zhu W, Kochan NA, The association of regional white matter lesions with cognition in a community-based cohort of older individuals. Neuroimage Clin 2018;19:14–21. doi: 10.1016/j.nicl.2018.03.035 30034997 PMC6051317

[96] Diagnostics Quest. Albumin test detail. In: Diagnostics Q, editor. online. online: Quest Diagnostics; 2019.

[97] Zahr NM. Peripheral tnfalpha elevations in abstinent alcoholics are associated with hepatitis c infection. PLoS One 2018;13:e0191586. doi: 10.1371/journal.pone.0191586 29408932 PMC5800541

[98] Piekarski D, Sullivan EV, Pfefferbaum A, Zahr NM. Poor subjective sleep predicts compromised quality of life but not cognitive impairment in abstinent individuals with alcohol use disorder. Alcohol 2022;103:37–43. doi: 10.1016/j.alcohol.2022.07.001 35870739 PMC9581497

[99] Shu L, Zhong K, Chen N, Gu W, Shang W, Liang J, Predicting the severity of white matter lesions among patients with cerebrovascular risk factors based on retinal images and clinical laboratory data: A deep learning study. Front Neurol 2023;14:1168836. doi: 10.3389/fneur.2023.1168836 37492851 PMC10363667

[100] Wei H, Liu D, Geng L, Liu Y, Wang H, Yan F. Application value of serum metabolic markers for cognitive prediction in elderly epilepsy. Neuropsychiatr Dis Treat 2022;18:2133–40. doi: 10.2147/ndt.S371751 36172266 PMC9512287

[101] Ma ZY, Wu YY, Cui HY, Yao GY, Bian H. Factors influencing post-stroke cognitive impairment in patients with type 2 diabetes mellitus. Clin Interv Aging 2022;17:653–64. doi: 10.2147/cia.S355242 35520948 PMC9063799

[102] Iwasaki M, Motokawa K, Watanabe Y, Hayakawa M, Mikami Y, Shirobe M, Nutritional status and body composition in cognitively impaired older persons living alone: The takashimadaira study. PLoS One 2021;16:e0260412. doi: 10.1371/journal.pone.0260412 34813604 PMC8610283

[103] Wang L, Wang F, Liu J, Zhang Q, Lei P. Inverse relationship between baseline serum albumin levels and risk of mild cognitive impairment in elderly: A seven-year retrospective cohort study. Tohoku J Exp Med 2018;246:51–7. doi: 10.1620/tjem.246.51 30249938

[104] Zhu JJ, Chen YJ, Chen LL, Zhao LJ, Zhou P. Factors that contribute to the cognitive impairment in elderly dialysis patients. Ther Apher Dial 2022;26:632–9. doi: 10.1111/1744-9987.13740 34550646

[105] Pei X, Lai S, He X, Masembe NP, Yuan H, Yong Z, Mild cognitive impairment in maintenance hemodialysis patients: A cross-sectional survey and cohort study. Clin Interv Aging 2019;14:27–32. doi: 10.2147/cia.S178854 30587951 PMC6304252

[106] Zhang YH, Yang ZK, Wang JW, Xiong ZY, Liao JL, Hao L, Cognitive changes in peritoneal dialysis patients: A multicenter prospective cohort study. Am J Kidney Dis 2018;72:691–700. doi: 10.1053/j.ajkd.2018.04.020 30007504

[107] Heaton RK, Franklin DR Jr, Deutsch R, Letendre S, Ellis RJ, Casaletto K, Neurocognitive change in the era of hiv combination antiretroviral therapy: The longitudinal charter study. Clinical Infectious Diseases 2015;60:473–80. 25362201 10.1093/cid/ciu862 PMC4303775

[108] Barokar J, McCutchan A, Deutsch R, Tang B, Cherner M, Bharti AR. Neurocognitive impairment is worse in hiv/hcv-coinfected individuals with liver dysfunction. J Neurovirol 2019;25:792–9. doi: 10.1007/s13365-019-00767-6 31281947 PMC6923581

[109] Parsons TD, Tucker KA, Hall CD, Robertson WT, Eron JJ, Fried MW, Neurocognitive functioning and haart in hiv and hepatitis c virus co-infection. AIDS 2006;20:1591–5. doi: 10.1097/01.aids.0000238404.16121.47 16868439

[110] Elliott C, Frith J, Day CP, Jones DE, Newton JL. Functional impairment in alcoholic liver disease and non-alcoholic fatty liver disease is significant and persists over 3 years of follow-up. Dig Dis Sci 2013;58:2383–91. doi: 10.1007/s10620-013-2657-2 23609794

[111] Horwich TB, Fonarow GC, Hamilton MA, MacLellan WR, Borenstein J. Anemia is associated with worse symptoms, greater impairment in functional capacity and a significant increase in mortality in patients with advanced heart failure. Journal of the American College of Cardiology 2002;39:1780–6. doi: 10.1016/S0735-1097(02)01854-5 12039491

[112] Frisbie JH. Anemia and hypoalbuminemia of chronic spinal cord injury: Prevalence and prognostic significance. Spinal Cord 2010;48:566–9. doi: 10.1038/sc.2009.163 19949419

[113] Gillum RF. The association between serum albumin and hdl and total cholesterol. J Natl Med Assoc 1993;85:290–2. 8478970 PMC2571905

[114] Haybar H, Pezeshki SMS, Saki N. Evaluation of complete blood count parameters in cardiovascular diseases: An early indicator of prognosis? Exp Mol Pathol 2019;110:104267. doi: 10.1016/j.yexmp.2019.104267 31194963

[115] Li B, Du B, Gu Z, Wu C, Tan Y, Song C, Correlations among peripheral blood markers, white matter hyperintensity, and cognitive function in patients with non-disabling ischemic cerebrovascular events. Frontiers in Aging Neuroscience 2022;14. doi: 10.3389/fnagi.2022.1023195 PMC9755852 36533171

[116] Godsland IF, North BV, Johnston DG. Simple indices of inflammation as predictors of death from cancer or cardiovascular disease in a prospective cohort after two decades of follow-up. Qjm 2011;104:387–94. doi: 10.1093/qjmed/hcq213 21106505

[117] Seo IH, Lee YJ. Usefulness of complete blood count (cbc) to assess cardiovascular and metabolic diseases in clinical settings: A comprehensive literature review. Biomedicines 2022;10. doi: 10.3390/biomedicines10112697 36359216 PMC9687310

[118] Weisberg HF. Osmotic pressure of the serum proteins. Ann Clin Lab Sci 1978;8:155–64. 345945

[119] Choi JW, Park JS, Lee CH. Genetically determined hypoalbuminemia as a risk factor for hypertension: Instrumental variable analysis. Sci Rep 2021;11:11290. doi: 10.1038/s41598-021-89775-3 34050200 PMC8163734

[120] Unger JK, Horn NA, Kashefi A, Blumberg A, Klosterhalfen B, Rossaint R. The influence of hypoalbuminemia on maximal flow rates and transmembrane pressure during plasmapheresis--an in vitro study. Blood Purif 2001;19:408–16. doi: 10.1159/000046972 11574738

[121] Gronewold J, Jokisch M, Schramm S, Himpfen H, Ginster T, Tenhagen I, Periventricular rather than deep white matter hyperintensities mediate effects of hypertension on cognitive performance in the population-based 1000brains study. J Hypertens 2022;40:2413–22. doi: 10.1097/hjh.0000000000003270 35983864 PMC9640292

[122] ten Dam VH, van den Heuvel DM, de Craen AJ, Bollen EL, Murray HM, Westendorp RG, Decline in total cerebral blood flow is linked with increase in periventricular but not deep white matter hyperintensities. Radiology 2007;243:198–203. doi: 10.1148/radiol.2431052111 17329688

